# A young man with secondary adrenal insufficiency due to empty sella syndrome

**DOI:** 10.1186/s12882-022-02699-6

**Published:** 2022-02-25

**Authors:** Hsi-Chih Chen, Chih-Chien Sung

**Affiliations:** Division of Nephrology, Department of Medicine, Tri-Service General Hospital, National Defense Medical Center, Taipei, Taiwan, R.O.C.

**Keywords:** Empty sella syndrome, Hyponatremia, Hypokalaemia, Corticosteroids

## Abstract

**Background:**

Empty sella syndrome is characterized by a constellation of symptoms that encompass various systems, and includes endocrine, neurologic, ophthalmologic, and psychiatric presentations. We here report a case of a young man presenting with severe hyponatremia due to empty sella syndrome and focus on changes in electrolytes during corticosteroid supplementation.

**Case report:**

A 36-year-old man presented with general weakness, poor appetite, and dizziness for 4 days. Physical assessment revealed lower limbs nonpitting oedema. Pertinent laboratory data showed severe hyponatremia (sodium 108 mmol/L). Endocrine work-up revealed low cortisol levels at 1.17 µg/dL (reference: 4.82–19.5 µg/dL) and inappropriately normal adrenocorticotropic hormone levels at 12.4 pg/mL (reference: 0.1–46.0 pg/mL), indicating secondary adrenal insufficiency. Brain magnetic resonance imaging confirmed the diagnosis of empty sella syndrome. He developed delirium and agitation one day after cortisol supplementation with a sodium correction rate of 10 mmol/L/day, while hypokalaemia (potassium 3.4 mmol/L) also developed. The symptoms improved after lowering the serum sodium level. This patient was eventually discharged after 12 days of hospitalization when the serum sodium and potassium levels were 139 mmol/L and 3.5 mmol/L, respectively.

**Conclusion:**

Herein, we address the importance of timely diagnosis of empty sella syndrome in patients with hyponatremia and highlight the close monitoring of the changes in electrolytes during corticosteroid replacement.

**Supplementary Information:**

The online version contains supplementary material available at 10.1186/s12882-022-02699-6.

## Background

Empty sella syndrome is characterized as herniation of the subarachnoid space within the pituitary fossa, which could have a variable degree of influence on the pituitary gland as well as adjacent bony structures [1]. As correlated with the localized mass effect, adjacent structures are usually affected by a reduction in size, as demonstrated by the commonly decreased size of the sphenoid sinus in previously published cases. Other typical features of empty sella syndrome include headache, irregular menses, excess weight, and visual disturbances [2]. The incidence of primary empty sella is approximately 11% in the normal population without any adjacent structure abnormalities [3]. The mechanism of empty sella-related hyponatremia could be attributed to endocrine abnormalities. Empty sella syndrome could present with hypopituitarism, including adrenal insufficiency, which is secondary to decreased stimulation by adrenocorticotrophic hormone. The consequence of hypocortisolism is failure to suppress vasopressin, leading to impaired free water excretion [4]. Electrolyte changes during management have rarely been discussed. We here present a case of empty sella syndrome initially presenting with severe hyponatremia and subsequent changes in serum potassium upon supplementation with cortisol, which is a distinguishing feature different from previously published cases in the current literature. There were no significant neurologic symptoms after timely reduction of serum sodium, and the patient was stably controlled in the outpatient department.

## Case presentation

**Fig. 1:**
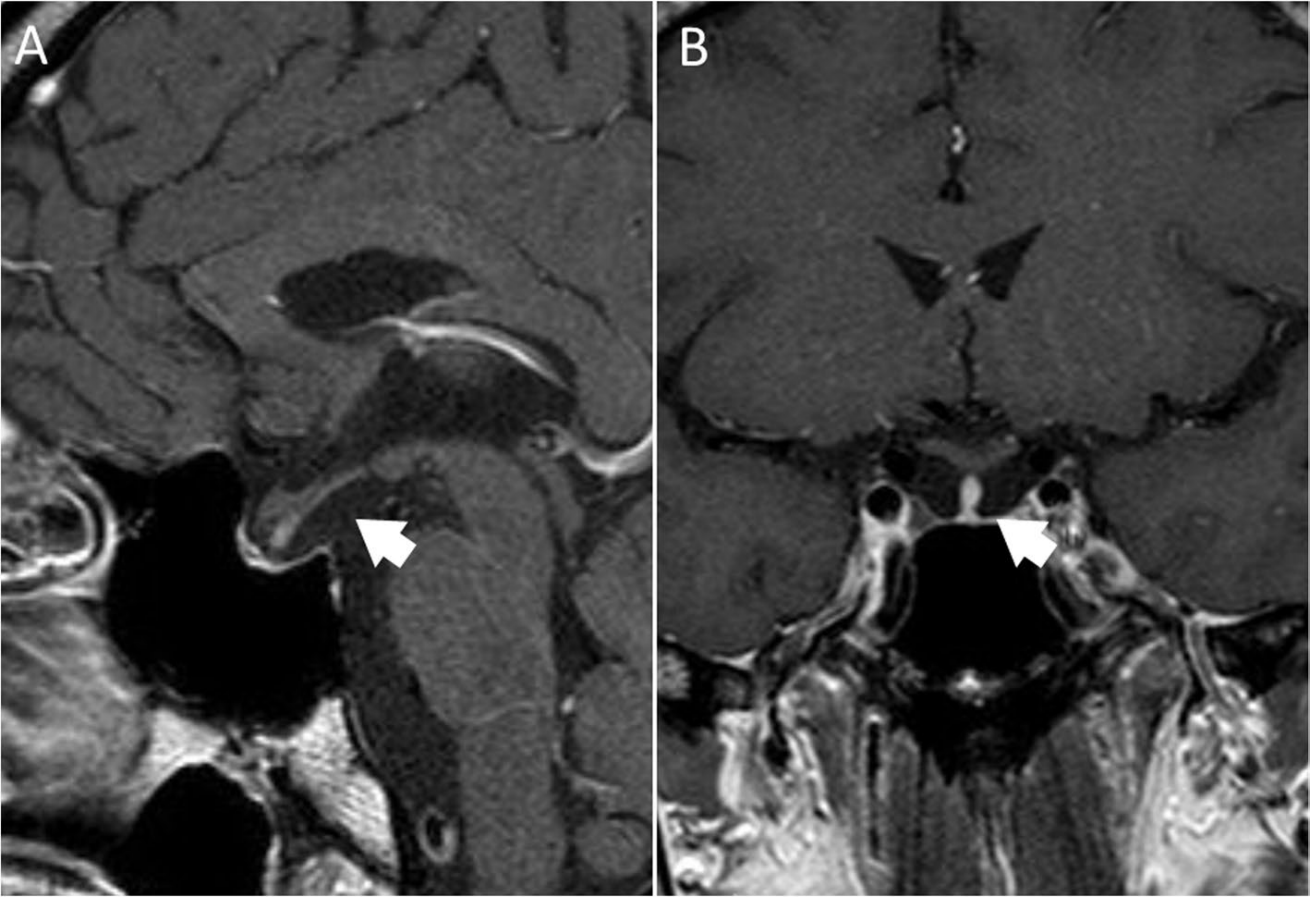
**A** MR T1-weighted sagittal image reveals cerebrospinal fluid filled into the pituitary fossa (*white arrow*). **B** MR T1-weighted coronal image showed a thin and flat pituitary gland (*white arrow*)

**Fig. 2:**
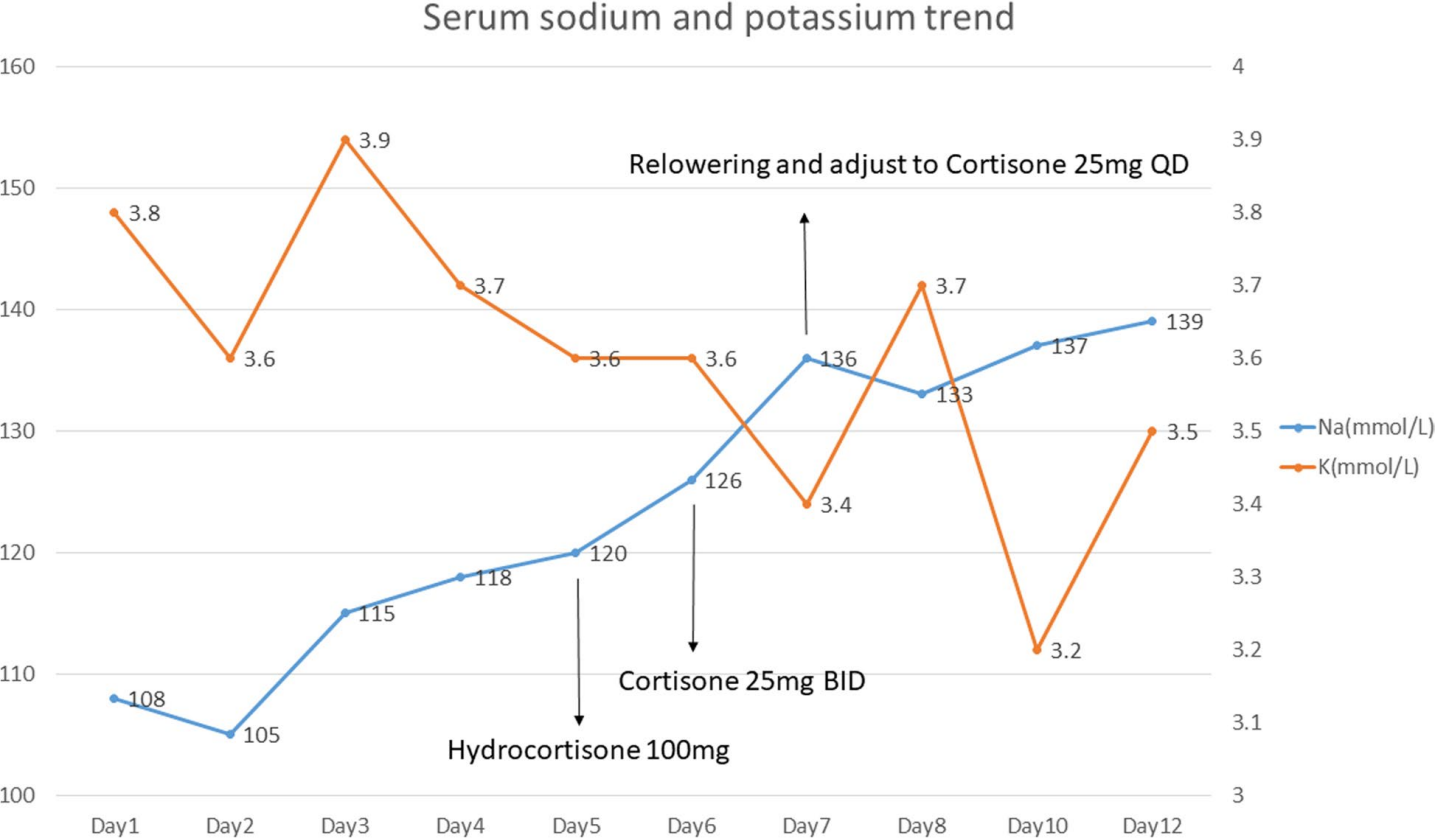
Serum sodium and potassium trends and the timing of cortisol replacement therapy during hospitalization

A 36-year-old man presented to the emergency department with general weakness, poor appetite, and dizziness for 4 days. He was noted to have hyponatremia (sodium 116 mmol/L) six years prior. Otherwise, there was no other medical, trauma, surgical, or recent medication history. He had normal growth and sexual development. In the emergency department, he was afebrile with a normal respiratory rate, pulse, and blood pressure. No headache, weight gain, or constipation was reported. Physical assessment revealed nonpitting oedema of the bilateral lower limbs. Pertinent laboratory investigations showed severe hyponatremia (sodium 108 mmol/L) with calculated serum osmolality 223 mOsm/kg H_2_O (reference: 275–295 mOsm/kg H_2_O), urine osmolality 804 mOsm/kg H_2_O (reference: 50–1200 mOsm/kg H_2_O), and urine sodium 130 mmol/L. Blood gas showed pH 7.357 and bicarbonate 24.2 mmol/L (reference: 22–27 mmol/L) with a relatively normal serum aldosterone level of 97.3 pg/ml (reference 12–150 pg/ml). During the initial management, the serum sodium level had a poor response to intravenous normal saline, and intravenous fluids were changed to hypertonic 3% saline two days after admission. Hormone assays revealed a low cortisol level at 1.17 µg/dL (reference range, 4.82–19.5 µg/dL), inappropriately normal adrenocorticotropic hormone at 12.4 pg/mL (reference: 0.1–46.0 pg/mL), low free T4 at 0.79 ng/dL (reference: 0.89–1.7 ng/dL), and inappropriately normal thyroid-stimulating hormone at 2.62 µIU/mL (reference: 0.25–5.00 µIU/mL). Testosterone was low at 0.72 ng/dL (reference: 249–836 ng/dL), along with low insulin-like growth factor-1 less than 15 ng/mL (reference: 57–241 ng/mL). Luteinizing hormone was 2.78 mIU/mL (reference: 1.7–8.6 mIU/mL), and prolactin was 5.6 ng/mL (reference: 4.04–15.2 ng/mL). The tentative diagnosis of secondary adrenal insufficiency was made according to the clinical and laboratory findings. Brain magnetic resonance imaging demonstrated a thin and flat pituitary gland located in the sella floor with prominent cerebral spinal fluid space (Fig. 1), compatible with empty sella syndrome. Intravenous hydrocortisone 100 mg was given once on the 5^th^ day of admission, followed by cortisone 25 mg twice daily starting on the 6^th^ day of admission. The serum sodium level rose from 126 to 136 mmol/L one day after cortisone administration (Fig. 2). Hypokalaemia (potassium 3.4 mmol/L) developed on the same day. Due to the concurrent presentation of delirium and agitation, the serum sodium levels were relowered. Symptoms gradually resolved after decreasing the serum sodium level to 133 mmol/L after administration of 5% dextrose solution. Potassium tablets were also prescribed at a dose of 5.08 mEq/day. The patient gradually recovered and returned to baseline health. He was discharged after 12 days of hospitalization. The patient was maintained on 25 mg cortisone daily without any sequelae when followed at the outpatient department.

## Discussion and conclusion

We present here a young man with secondary adrenal insufficiency due to empty sella syndrome who developed symptoms of delirium without overcorrection of sodium. Of note, hypokalaemia also developed during cortisol supplementation. Although empty sella syndrome has been well discussed in the literature, changes in electrolytes such as sodium and potassium during corticosteroid supplementation are easily overlooked in clinical practice. Herein, we focus on timely recognition of hyponatremia as an early sign of empty sella syndrome with adrenal insufficiency and the effects of corticosteroids on potassium during management.

Empty sella syndrome is a neuroendocrine disorder and can be divided into primary (also called arachonidocele) and secondary empty sella syndrome [2]. Primary empty sella syndrome may be related to congenital structural abnormalities. Secondary causes could be surgery, radiotherapy, drugs, trauma, or autoimmune disorders. Most patients are asymptomatic, but some patients develop panhypopituitarism [5]. Panhypopituitarism is the most common hormone disorder, while other aspects of pituitary deficiency include secondary adrenal insufficiency, hypothyroidism, hypogonadism, and growth hormone deficiency [6]. Secondary adrenal insufficiency is caused by reduced secretion of adrenocorticotrophic hormone, resulting in hypocortisolism [4]. Hyponatraemia is the result of excessive vasopressin secretion due to hypocortisolism that fails to suppress vasopressin [4, 7]. Instead of the usual hyponatremia management of fluid restriction, treatment of empty sella syndrome-associated hyponatremia is encompassed by glucocorticoid supplement and sodium replacement [8]. If patients with empty sella syndrome have concomitant central hypothyroidism and adrenal insufficiency, clinicians should keep in mind that the restoration of euthyroidism with levothyroxine therapy might precipitate an adrenal crisis in patients with unrecognized central hypoadrenalism, as normalization of thyroid function increases cortisol metabolism in these patients, thereby leading to a greater glucocorticoid requirement [9]. It has also been reported that patients with hyponatremia related to adrenocorticotrophic hormone deficiency may have relative hypoaldosteronism and metabolic acidosis, but our patient did not present with metabolic acidosis initially and had relatively normal aldosterone levels [10, 11]. This could be explained by the relative hypovolemic status due to his poor intake and vomiting.

In management of hyponatremia, the sodium correction rate is suggested to be less than 12 mmol/L/day or even more restricted to 8 to 10 mmol/L/day in recent guidelines, and hypokalaemia has been recognized as an important risk factor for developing osmotic demyelination syndrome [4, 12, 13]. Since corticosteroid replacement is the mainstay treatment to manage secondary adrenal insufficiency-associated hyponatremia, it is crucial to be cautious not to correct sodium levels rapidly when combining corticosteroids, as the synergistic effect of corticosteroid replacement along with intravenous sodium administration could result in rapidly downregulated vasopressin [14]. Nevertheless, this patient presented with delirium and agitation without overcorrection of sodium after corticosteroid treatment, and decreased serum potassium was noted. A decrease in Na^+^‑K^+^‑ATPase activity during hypokalaemia may affect the cell’s ability to preserve its volume and predispose the cell to injury by rapid osmotic stress [15]. Once the clinical clues of overcorrection of sodium develop, immediate discontinuation of sodium correction and relowering of serum sodium are necessary. The patient’s symptoms were alleviated after immediate relowering of sodium and supplementation with potassium.

Corticosteroids, including fludrocortisone, hydrocortisone, and prednisolone, also have mineralocorticoid effects resulting in sodium and water retention, potassium excretion, and transcellular potassium shift caused by hyperinsulinaemia [16, 17]. A decrease in serum potassium level might also arise from the mineralocorticoid effect, which could be a hint of the risk of rapid sodium correction. Therefore, monitoring serum potassium closely in addition to the sodium correction rate is highly recommended in patients receiving corticosteroid and intravenous sodium supplementation.

In conclusion, electrolyte imbalance, such as severe hyponatremia, could be the sole presenting sign of empty sella syndrome. Clinicians should be aware of the risks of hypokalaemia and sodium overcorrection rate during the management of adrenal insufficiency. Careful monitoring of the changes in electrolytes when combining corticosteroid therapy should be emphasized in patients with secondary adrenal insufficiency caused by empty sella syndrome to avoid any neurological deficit.

## Supplementary Information

**Additional file 1.**

**Additional file 2.**

**Additional file 3.**

## Data Availability

The datasets analysed during the current study care available from the corresponding author on reasonable request.
