# Supplementary material for: A young man with secondary adrenal insufficiency due to empty sella syndrome
Source: BMC Nephrol. 2022 Feb 25;23:81. doi: 10.1186/s12882-022-02699-6 (PMC8876128; doi:10.1186/s12882-022-02699-6)
Supplement: Supplementary file 3 — Additional file 3. [file 12882_2022_2699_MOESM3_ESM.pdf]

AJE®

Editing Certificate

This document certifies that the manuscript

A Young Man with Secondary Adrenal Insufficiency due to Empty Sella Syndrome

prepared by the authors

Hsi-Chih Chen, Chih-Chien Sung

was edited for proper English language, grammar, punctuation, spelling, and overall style by one or more of the highly qualified native English speaking editors at AJE.

This certificate was issued on **July 28, 2021** and may be verified on the AJE website using the verification code **9840-339A-849F-307F-23D8** .

AJE® EDITING CERTIFIED

Neither the research content nor the authors' intentions were altered in any way during the editing process. Documents receiving this certification should be English-ready for publication; however, the author has the ability to accept or reject our suggestions and changes. To verify the final AJE edited version, please visit our verification page at aje.com/certificate. If you have any questions or concerns about this edited document, please contact AJE at support@aje.com.

AJE provides a range of editing, translation, and manuscript services for researchers and publishers around the world. For more information about our company, services, and partner discounts, please visit aje.com.
