# Supplementary material for: A young man with secondary adrenal insufficiency due to empty sella syndrome
Source: BMC Nephrol. 2022 Feb 25;23:81. doi: 10.1186/s12882-022-02699-6 (PMC8876128; doi:10.1186/s12882-022-02699-6)
Supplement: Supplementary file 2 — Additional file 2. [file 12882_2022_2699_MOESM2_ESM.docx]

**2^nd^ Aug 2021**

Editor-in-Chief, BMC Nephrology

**Hayley Henderson,**

Locum Editor, BMC Nephrology

**Catherine Rice,**

Dear Editor-in-Chief Prof. Henderson and Locum Editor Prof. Rice,

Thank you for considering our manuscript **(BNEP-D-20-00980)** entitled **“A Young Man with Secondary Adrenal Insufficiency Due to Empty Sella Syndrome”** by Sung CC et al for publication as a for publication as a case report in ***BMC Nephrology.***

We are extremely grateful to you and the reviewers for the constructive critique of our manuscript. We have responded to the reviewer’s comments on separate sheets and careful attention to each of the suggestions has led to a significant improvement in our article. All the changes are highlighted in red font and underlined in the revised manuscript. Therefore, we resubmitted our revised version to ***BMC Nephrology*** according to the guideline to the authors.

We look forward to your prompt reply.

Yours sincerely,

Chih-Chien Sung, M.D, PhD

Division of Nephrology, Department of Medicine

Tri-Service General Hospital

No 325, Section 2, Cheng-Kung Road,

Neihu 114, Taipei, Taiwan.

Tel: 886-2-87927213 Fax: 886-2-87927322

E-mail: sungchihchien@gmail.com

**Response to Editor:**

**Point 1. We recommend that you ask a native English-speaking colleague to help you copyedit the paper. If this is not possible, you may need to use a professional language editing service.**

**Remedy:** We are grateful for your suggestion. The revised manuscript has been edited by professional English editing before re-submission. All the changes are highlighted in red font and underlined in the revised manuscript. The editing certificate is shown below.

**Response to Reviewer 1:**

**Point 1. SNa was 108 mEq/l and Serum osmolality 255, value not expected, give a comment.
Remedy:** We greatly appreciate your valuable comment and point out this question. Serum osmolality was 255 mOsm/kgH_2_0 when the serum sodium was 120 mmol/L on day 5 after admission. We apologized that the serum osmolality was not measured when serum sodium was 108 mEq/L, but the calculated serum osmolality was 223 mOsm/kgH_2_0. We have revised it according to this valuable suggestion, and please see **line 84 on page 5.**

**Point 2. Urine osmolality was 804, reference is not exact (50-1100)**
**Remedy:** Thanks for pointing out this typo of reference range. The correct urine osmolality reference range is 50-1200 mOsm/kgH_2_O and we have revised it on the revised manuscript. Please see **line 85 on page 5.**

**Point 3. The authors don't report bicarbonate! this must be given (frequently low in patients with SIADH secondary to panhypopituitarism) a comment on this must be added**

**Remedy:** We are grateful for the valuable comment. Yes, it has been reported that patients with hyponatremia related to ACTH deficiency and confirms a decrease in total CO_2_ levels related to respiratory alkalosis and to relative hypoaldosteronism (Please see **Ref. 1**). However, this patient had pH 7.357, bicarbonate 24.2 mmol/L, and the partial pressure of carbon dioxide 44.2 mmHg (reference, 38.0-49.0 mmHg). Furthermore, the serum aldosterone level for this patient is 97.3 pg/ml (reference 12-150 pg.ml) with plasma renin activity of 0.30 ng/ml/hr (reference 0.15-2.33 ng/ml/hr). Our patient did not present with metabolic acidosis initially with relative normal aldosterone level, and it could be explained by relative hypovolemic status due to his poor intake and vomiting. In the **Ref. 1**, two patients with ACTH deficiency still had normal range of bicarbonate. Therefore, the acid-base condition depends on the aldosterone level that will be influenced by fluid status of patients. Thanks for your valuable comment and we have provided the blood gas value and aldosterone level **on page 5, line 85-87** as well as comment **“**It has also been reported that patients with hyponatremia related to adrenocorticotrophic hormone deficiency may have relative hypoaldosteronism and metabolic acidosis, but our patient did not present with metabolic acidosis initially and had relatively normal aldosterone levels (10, 11). This could be explained by the relative hypovolemic status due to his poor intake and vomiting**.”** in the discussion section **on page 8, line 137-141.**

**Reference**

1. Decaux G, Musch W, Penninckx R, Soupart A. Low plasma bicarbonate level in hyponatremia related to adrenocorticotropin deficiency. J Clin Endocrinol Metab. 2003 Nov;88(11):5255-7. doi: 10.1210/jc.2003-030399. PMID: 14602758.

Lastly, I am deeply honored by the time and effort you spent in reviewing this manuscript. In reviewing and revising our manuscript, I was motivated to read more and thus learn more from your criticisms.

**Response to Reviewer 2.**

**Point 1 RELEVANCE - Does this case report make a contribution to medical knowledge, have educational value, or highlight the need for a change in clinical practice or diagnostic/prognostic approaches?
No, the findings of this report are well known and/or similar reports have already been published
Remedy:** We appreciate this insightful comment. According to your suggestion, we have revised our manuscript with more medical education in the discussion section and all the changes were shown with red font and underlined. In the revised manuscript, we present here a young man with secondary adrenal insufficiency due to empty sella syndrome who developed symptoms of delirium without overcorrection of sodium. Of note, hypokalaemia also developed during cortisol supplementation. Although empty sella syndrome has been well discussed in the literature, changes in electrolytes such as sodium and potassium during corticosteroid supplementation are easily overlooked in clinical practice. Herein, we focus on timely recognition of hyponatremia as an early sign of empty sella syndrome with adrenal insufficiency and the effects of corticosteroids on potassium during management. **Please see on page 7, line 114-120.** We also provide some education point about central hypothyroidism and hypoaldosteronism that may be relate to this disease in the discussion section and please see **page 8 on line 132-141** and hope it will provide insightful educational value for readers. Other changes are highlighted in red font and underlined in the revised manuscript.

**Point 2 CASE DESCRIPTION - Are the details of the case sufficiently well described to understand the patient's symptoms and course of treatment?
No - there are minor issues**
**Remedy:** Thanks for these valuable comments, and we have revised case presentation section of the manuscript for readers to understand the patient's symptoms and course of treatment clearly. Furthermore, the revised manuscript has been edited by professional English editing before re-submission. Please see the changes are highlighted in red font and underlined on the section of case presentation in this revised manuscript. **Please see line 78-94 on page 5 and line 95-108 on page 6.** The editing certificate is shown below.

**Point 3** **DIAGNOSIS/INTERPRETATION - Based on the facts presented, are the diagnosis, interpretation, and course of treatment medically sound?**
Yes, the work described is medically sound
**Remedy:** Thank you.

**Point 4 DISCUSSION OF THE CASE - Does the discussion appropriately analyze the importance of the findings and their relevance to future understanding of disease processes, diagnosis or treatment? Has an adequate literature review pertinent to the case been included?
No - there are minor issues**
**Remedy:** We are grateful for this valuable comment for discussion section. In discussion, we revised our first paragraph to summarize this report and try to make it clear to readers, and please see **Please see on page 7, line 114-120.** In addition to **t**he pathophysiology of empty sella syndrome and associated endocrine disorder were addressed according to new cited references (9, 10, 11), we further discussed central hypothyroidism and hypoaldosteronism that may be related to this disease in the discussion section and please see **page 8 on line 132-141** and also point out the possible risk factors during the correction of sodium level and restoring the cortisol level in 2^nd^ and 3^rd^ paragraphs in the discussion section, and new references (13, 15) were also cited in the revised manuscript. All the changes were highlighted in red font and underlined in the discussion section. **Please see on page 7, 8, and 9.**

**Reference (***the reference number is according to the reference number shown in the manuscrip*t)

9.	Beck-Peccoz P, Rodari G, Giavoli C, Lania A. Central hypothyroidism - a neglected thyroid disorder. Nat Rev Endocrinol. 2017;13(10):588-98.

10.	Schneider EG, Taylor RE Jr, Radke KJ, Davis PG. Effect of sodium concentration on aldosterone secretion by isolated perfused canine adrenal glands. Endocrinology. 1984 Dec;115(6):2195-204.

11.	Decaux G, Musch W, Penninckx R, Soupart A. Low plasma bicarbonate level in hyponatremia related to adrenocorticotropin deficiency. J Clin Endocrinol Metab. 2003 Nov;88(11):5255-7.

12.	Sterns RH, Riggs JE, Schochet SS Jr. Osmotic demyelination syndrome following correction of hyponatremia. N Engl J Med. 1986 Jun 12;314(24):1535-42.

13.	George JC, Zafar W, Bucaloiu ID, Chang AR. Risk Factors and Outcomes of Rapid Correction of Severe Hyponatremia. Clin J Am Soc Nephrol. 2018 Jul 6;13(7):984-992.

15.	Lohr JW. Osmotic demyelination syndrome following correction of hyponatremia: association with hypokalaemia. Am J Med. 1994;96(5):408-13.

**Point 5 OVERALL MANUSCRIPT POTENTIAL - Could an appropriately REVISED version of this work represent a technically sound contribution?
No - manuscript has some fundamental flaw(s)**
**Remedy:** Thank you and I am deeply honored by the time and effort you spent in reviewing this manuscript. We have responded to your comments point to point and careful attention to each of the suggestions has led to a significant improvement in our revised manuscript. In the case presentation and discussion section, we try our best to provide more informative and educational points for readers or researcher to understand the possible risks in patients with secondary adrenal insufficiency due to empty sella. In addition, the revised manuscript was also edited by professional English editing. All the changes are highlighted in red font and underlined in the revised manuscript. We hope our revised manuscript sufficient novelty in education for clinician. Lastly, we really appreciate your review, and we were motivated to read more and thus learn more from your criticisms

Lastly, I am deeply honored by the time and effort you spent in reviewing this manuscript. In reviewing and revising our manuscript, I was motivated to read more and thus learn more from your criticisms.

**Response to Reviewer 3.**

**Point 1. PEER REVIEWER COMMENTS:**
GENERAL COMMENTS: This is an interesting case report, however, there is no novelty reported. Additionally, the central hypothyroidism is not completely discussed and LT4 replacement may also impact sodium behavior.
**Remedy**: We appreciate your detailed review and valuable comment. Although our case has low free T4 at 0.79 ng/dL (reference: 0.89-1.7 ng/dL), and inappropriately normal thyroid-stimulating hormone at 2.62 µIU/mL (reference: 0.25-5.00 µIU/mL), but we did not prescribe levothyroxine for him during the supplement of cortisol. We totally agree with your point and we have revised the discussion section **on page 8, line 132-137** according to this valuable suggestion “ the concomitant central hypothyroidism and adrenal insufficiency were discussed in our revised manuscript: If patients with empty sella syndrome have concomitant central hypothyroidism and adrenal insufficiency, clinicians should keep in mind that the restoration of euthyroidism with levothyroxine therapy might precipitate an adrenal crisis in patients with unrecognized central hypoadrenalism, as normalization of thyroid function increases cortisol metabolism in these patients, thereby leading to a greater glucocorticoid requirement.” Please see the changes, highlighted in red font and underlined, on the section of discussion and conclusion in this revised manuscript.

**Point 2** **What did you find to be the major findings of the manuscript?**
This manuscript describes a patient with secondary adrenal insufficiency due to empty sella syndrome. The patient developed neurological symptoms after treatment
**Remedy:** Thank you for your review and summary of our finding of this case report.

**Point 3** What unusual or novel methods does this paper use, if any?
None.
**Remedy:** Thank you and we try our best to make this case report better and provide insightful education for readers. Some response was shown in point 4, and please see remedy of point 4.

**Point 4 How does this work contribute to the field?**
There is no minor or major contribution to the field
**Remedy:** We appreciate this comment. According to your suggestion, we have revised our manuscript, especially in case presentation and discussion sections. In case presentation section, we have revised it for readers to understand the patient's symptoms and course of treatment clearly and send this manuscript for professional English editing before re-submission. Please see the certificate below and **see line 78-94 on page 5 and line 95-108 on page 6.**

In discussion section, we revised our first paragraph to summarize this report and try to make it clear to readers, and please see **Please see on page 7, line 114-120.** In addition to **t**he pathophysiology of empty sella syndrome and associated endocrine disorder were addressed according to new cited references (9, 10, 11), we further discussed central hypothyroidism and hypoaldosteronism that may be relate to this disease in the discussion section and please see **page 8 on line 132-141** and also point out the possible risk factors during the correction of sodium level and restoring the cortisol level in 2^nd^ and 3^rd^ paragraphs in the discussion section, and new references (13, 15) were also cited in the revised manuscript. All the changes were highlighted in red font and underlined in the discussion section. **Please see on page 7, 8, and 9.**

**Reference (***the reference number is according to the reference number shown in the manuscrip*t)

9.	Beck-Peccoz P, Rodari G, Giavoli C, Lania A. Central hypothyroidism - a neglected thyroid disorder. Nat Rev Endocrinol. 2017;13(10):588-98.

10.	Schneider EG, Taylor RE Jr, Radke KJ, Davis PG. Effect of sodium concentration on aldosterone secretion by isolated perfused canine adrenal glands. Endocrinology. 1984 Dec;115(6):2195-204.

11.	Decaux G, Musch W, Penninckx R, Soupart A. Low plasma bicarbonate level in hyponatremia related to adrenocorticotropin deficiency. J Clin Endocrinol Metab. 2003 Nov;88(11):5255-7.

12.	Sterns RH, Riggs JE, Schochet SS Jr. Osmotic demyelination syndrome following correction of hyponatremia. N Engl J Med. 1986 Jun 12;314(24):1535-42.

13.	George JC, Zafar W, Bucaloiu ID, Chang AR. Risk Factors and Outcomes of Rapid Correction of Severe Hyponatremia. Clin J Am Soc Nephrol. 2018 Jul 6;13(7):984-992.

15.	Lohr JW. Osmotic demyelination syndrome following correction of hyponatremia: association with hypokalaemia. Am J Med. 1994;96(5):408-13.

Herein, we focus on timely recognition of hyponatremia as an early sign of empty sella syndrome with adrenal insufficiency and the effects of corticosteroids on potassium during management. All changes are highlighted in red font and underlined in the revised manuscript. We hope our revised manuscript could contribute to the field. Thank you.

**Point 5** **How could other researchers use the information presented in this paper in their own work?**
I can't see useful information in this work. nor for the clinician neither for the scientist
**Remedy:** We appreciate this valuable comment. According to your suggestion, we have revised our manuscript with more informative medical education in the discussion section, and all the changes were shown with red font and underlined. In the 1^st^ paragraph of this revised manuscript, we summarize that” we present here a young man with secondary adrenal insufficiency due to empty sella syndrome who developed symptoms of delirium without overcorrection of sodium. Of note, hypokalaemia also developed during cortisol supplementation. Although empty sella syndrome has been well discussed in the literature, changes in electrolytes such as sodium and potassium during corticosteroid supplementation are easily overlooked in clinical practice. Herein, we focus on timely recognition of hyponatremia as an early sign of empty sella syndrome with adrenal insufficiency and the effects of corticosteroids on potassium during management.” **Please see on page 7, line 114-120.** Furthermore, we also provide some education point about central hypothyroidism and hypoaldosteronism and the change of electrolytes during management of adrenal insufficiency in the discussion section and please see **page 8 on line 132-141** and hope it will provide informative educational value for readers and researchers.

**Point 6** **REQUESTED REVISIONS:**
I do not find in this case report sufficient novelty or a new side effect.

**Remedy:** Thank you for pointing out this important viewpoint. I am deeply honored by the time and effort you spent in reviewing this manuscript. In this revised manuscript, we have responded to your comments point to point and careful attention to each of the suggestions has led to a significant improvement in our revised manuscript. In the case presentation and discussion section, we try our best to provide more informative and educational points for readers or researcher to understand the possible risks in patients with secondary adrenal insufficiency due to empty sella. In addition, the revised manuscript was also edited by professional English editing. All the changes are highlighted in red font and underlined in the revised manuscript. We hope our revised manuscript sufficient novelty in education for clinician. Lastly, we really appreciate your review, and we were motivated to read more and thus learn more from your criticisms
